# Amphetamine-Dextroamphetamine-Induced Cardiomyopathy: A Case Report on Heart Failure in a Young Addict

**DOI:** 10.7759/cureus.71044

**Published:** 2024-10-07

**Authors:** Abdulkreem Al-Juhani, Eman M Alyaseen, Saeed Aseri, Omar M Alghamdi

**Affiliations:** 1 Surgery, Faculty of Medicine, King Abdulaziz University, Jeddah, SAU; 2 College of Medicine and Medical Science, Arabian Gulf University, Manama, BHR; 3 Medicine, Faculty of Medicine, King Abdulaziz University, Jeddah, SAU; 4 Cardiology, Althager General Hospital, Jeddah, SAU

**Keywords:** amphetamine-dextroamphetamine, amphetamine-induced cardiomyopathy, cardiomyopathy, medication overuse, narcolepsy treatment, prescribed adderall

## Abstract

In teenagers and young adults, dextroamphetamine-induced cardiomyopathy is becoming a more common cause of heart failure. More patients are at risk of this cardiac manifestation due to the medication's increased use in the treatment of attention-deficit/hyperactivity disorder (ADHD), particularly those who are unaware of the risks associated with using it improperly to improve concentration for poor performance. It becomes imperative to draw attention to the link between heart failure and amphetamine-dextroamphetamine usage to raise awareness of this illness. This case study revolves around a 32-year-old man with a complicated medical history who was recently diagnosed with heart failure with reduced ejection fraction (HFrEF < 10%), which was linked to amphetamine-induced cardiomyopathy. His recent diagnosis of ADHD and the treatment that followed raise questions regarding the potential for drug-induced worsening of underlying cardiomyopathy. To address his cardiac and concomitant issues, the patient is currently on a complete pharmaceutical regimen that includes atorvastatin, aspirin, furosemide, spironolactone, enoxaparin, omeprazole, and carvedilol. To maximize results and stop additional cardiac deterioration, patients with dual diagnoses, especially those involving amphetamine use and preexisting cardiac conditions, need to be carefully monitored and managed by a multidisciplinary team.

## Introduction

The existence of left ventricular (LV) systolic failure without aberrant loading conditions (such as valve disease or arterial hypertension) or coronary artery disease severe enough to compromise global systolic function is known as dilated cardiomyopathy [1]. It is a common condition that accounts for 40%-50% of heart failure occurrences. Right ventricular dilatation and dysfunction may be present although they are not required for the diagnosis. Chamber dilation caused by ventricular remodeling, along with normal or reduced wall thickness and a reduction in systolic function, are the hallmarks of dilated cardiomyopathy [2].

Stimulant illicit substances, such as amphetamine-dextroamphetamine, have been linked to cardiovascular consequences via catecholamine-mediated mechanisms, including tachycardia, vasoconstriction, vasospasm, hypertension, and direct cardiac damage [3]. An unusual but essential and developing problem of frequent amphetamine-dextroamphetamine use is the development of nonischemic cardiomyopathy in young adults, which may be caused by tachycardia and progress to heart failure [4]. This is an increasingly common occurrence in young adults with heart failure who do not have any other risk factors. The significance of this occurrence stems not only from the medication's widespread misuse, which puts many teenagers and young adults at risk for nonischemic cardiomyopathy and mortality but also from the need to outline various options for managing and following up on patients with psychiatric illnesses who do require treatment with this medication. It is important to remember that as the medication's use is discussed, the cardiac consequences must be addressed and presented. The case of a 32-year-old man with a three-month history of amphetamine-dextroamphetamine usage and heart failure engendered by nonischemic cardiomyopathy is presented in this study.

## Case presentation

The patient is a 32-year-old male with a known history of cardiomyopathy, evidenced by heart failure with reduced ejection fraction (HFrEF < 10%). He presented to the Emergency Department (ER) with a four-day history of shortness of breath (SOB), orthopnea, lower limb swelling, and abdominal distention. Notably, there was an absence of chest pain, palpitations, vertigo, loss of consciousness, fever, vomiting, or nausea. Additionally, he reported no history of abdominal pain, changes in bowel habits, or urinary symptoms. One year ago, the patient was diagnosed at a tertiary hospital with HFrEF due to amphetamine-induced cardiomyopathy, following a three-month history of ADHD diagnosis. His social history included smoking one pack of cigarettes per day, and he denied any use of alcohol, recreational drugs (such as cocaine), or intravenous drug use. On physical examination, the patient was found to be in distress, with jugular venous distention, bilateral pitting edema, and crackles on auscultation of the lungs. At the initial evaluation cardiac examination revealed an irregularly irregular rhythm, displaced apical impulse, and an S3 heart sound. The patient's extremities were cool to the touch, with diminished peripheral pulses, suggesting severe decompensated heart failure. He is currently under regular follow-up, with plans for the insertion of an Implantable cardioverter defibrillator (ICD). His current medication regimen includes Entresto, carvedilol, spironolactone, furosemide, atorvastatin, aspirin, and omeprazole. He was recently admitted to the ward for management of acute decompensated heart failure (ADHF).

**Figure 1:**
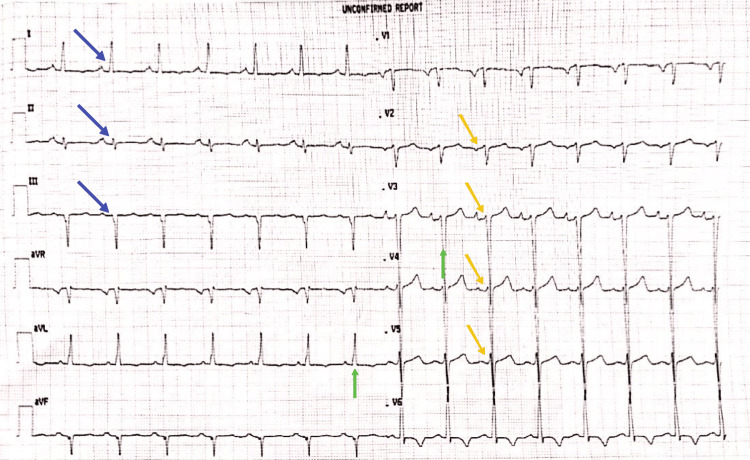
An ECG showing sinus rhythm, left axis deviation (blue arrow), left ventricular hypertrophy (green arrow), and poor R-wave progression (yellow arrow). ECG, electrocardiogram

After stabilizing the patient, tachycardia with a heart rate of 133 and a regular rhythm was observed during the first consultation with the treating team, who evaluated him for palpitations, previously confirmed cardiomyopathy, and tachycardia. No whispers were welcomed. He experienced non-pitting bilateral lower extremities edema and fine rales in the midlung and bases on both sides. An ECG revealed sinus rhythm, left axis deviation, LV hypertrophy, and poor R progression (Figure 1).

He is currently taking the following medications: Atorvastatin 20 mg PO OD, Aspirin 81 mg PO OD, Furosemide 40 mg IV BID, Spironolactone 25 mg PO OD, Enoxaparin 40 mg SC OD, Omeprazole 40 mg IV OD, and Carvedilol 6.25 mg PO BID. As Table 1 shows, thyroid-stimulating hormone and a lipid panel were also included in the evaluation, which were normal. Following this, a stress transthoracic echocardiogram (TTE) showed an LV ejection fraction of 8%-9%, a diastolic LV internal diameter (LVID) of 4.5 cm (normal 3.5-5.6 cm), and a systolic LVID of 4.4 cm (normal 2.0-4.0 cm), all of which were elevated and suggested a higher risk of heart failure. After a 48-hour Holter monitor, the results showed no discernible diurnal changes, infrequent ventricular and supraventricular ectopy with no noticeable pauses, and sinus rhythm with rates ranging from 42 to 106 beats per minute (bpm), with an average of 59 bpm. He then had a left cardiac catheterization, which confirmed the diagnosis of nonischemic cardiomyopathy by showing angiographically normal coronary arteries. Subsequently, amphetamine-dextroamphetamine was stopped. An implanted ICD was taken into consideration since, as will be further explained later, certain individuals with methamphetamine-induced cardiomyopathy recover by simply discontinuing. The patient did show some improvement in his symptoms during the one-month follow-up on the medication.

**Table 1:** Summary of the laboratory and diagnostic test results with normal ranges and interpretation.

Investigation	Result	Normal range	Interpretation
Thyroid-stimulating hormone (TSH)	2.5 mIU/L	0.4-4.0 mIU/L	Within normal range
Lipid panel			
Low-density lipoprotein (LDL)	100 mg/dL	<100 mg/dL	Borderline high
High-density lipoprotein (HDL)	55 mg/dL	≥40 mg/dL	Normal
Triglycerides	140 mg/dL	<150 mg/dL	Normal
Total cholesterol	180 mg/dL	<200 mg/dL	Normal
Left ventricular ejection fraction	8%-9%	50%-70%	Severely reduced, indicating heart failure
Diastolic left ventricular internal diameter (LVID)	4.5 cm	3.5-5.6 cm	Within normal range
Systolic left ventricular internal diameter (LVID)	4.4 cm	2.0-4.0 cm	Elevated, suggesting systolic dysfunction
48-hour Holter monitor	Sinus rhythm, 42-106 bpm	60-100 bpm (resting)	Slight bradycardia with sinus rhythm
Average heart rate (Holter monitor)	59 bpm	60-100 bpm (resting)	Slight bradycardia

## Discussion

Cardiac toxicity from amphetamine use was first described in the mid-1970s, and it is a recognized entity of heart failure in the modern era, particularly in the younger population, with mean diagnostic age at 40 ± 10 years, and male predominance (83%) [5]. The average duration of methamphetamine use before a diagnosis of congestive heart failure is five years, with almost half (18%) of the reported diagnoses made in the first 12 months [6]. Amphetamines are synthetic stimulants derived from the core structure of 𝛽-phenylethylamine, they exert their effects on the cardiovascular system primarily through the release of monoamine (norepinephrine and dopamine) within the synapses of the peripheral and central nervous system along with inhibition of their metabolism. This results in increased heart rate, blood pressure, vasoconstriction, and vasospasm. While tachycardia and hypertension are associated with increased oxygen demand, vasoconstriction and vasospasm decrease cardiac oxygen supply, which further contributes to the development of cardiomyopathy. Additionally, persistent catecholamine exposure or rapid intravenous infusion can result in cardiotoxic effects with apoptosis or necrosis of cardiomyocytes. These cardiotoxic effects include electrical and structural cardiac remodeling such as myocardial contractility changes, myocardial fibrosis, and dilated cardiomyopathy, with lymphocytic infiltrates [7,8].

Clinically, patients with amphetamine-induced cardiomyopathy present with symptoms consistent with heart failure, such as dyspnea, orthopnea, paroxysmal nocturnal dyspnea, and peripheral edema, as seen in this case. Moreover, patients may present with other symptoms including chest pain, tachycardia, and palpitation [9]. A retrospective study conducted by Kueh et al. shows that echocardiographic findings often reveal significant LV dilation (mean LV end-diastolic dimension [LVEDD], 6.8 ± 1.0 cm; mean LV end-systolic dimension [LVESD], 6.1 ± 1.2 cm) and reduced systolic function. Significant functional mitral regurgitation was documented in 33.3% of patients, which are hallmark of dilated cardiomyopathy. Additionally, the presence of tachycardia, as noted in this patient, is a common feature due to the sympathomimetic effects of amphetamines. Cardiac biomarker troponin and natriuretic-peptide (BNP) levels were elevated in most of the patients along with electrocardiogram (ECG) changes [7]. Amphetamine-induced cardiomyopathy is typically nonischemic, as was confirmed in this patient through left cardiac catheterization, which showed normal coronary arteries. The absence of coronary artery disease further supports the diagnosis of a toxic, nonischemic form of cardiomyopathy.

Guidelines for methamphetamine-associated cardiomyopathy treatment have not been established yet. An end to amphetamine-dextroamphetamine abuse is the cornerstone of treatment ceasing the drugs causes improvements in the patient's condition; according to some studies, the LVEF can improve by 7% in as little as seven days [10]. In some instances, the severity of cardiomyopathy changes eventually leads to decompensated heart failure that requires heart transplantation for continued survival [11]. In our case, amphetamine therapy was discontinued, as well as standard heart failure therapy, including the use of ACE inhibitors, beta-blockers, and diuretics. This patient was appropriately managed with a comprehensive pharmacological regimen, including Entresto (sacubitril/valsartan), carvedilol, spironolactone, and furosemide, all of which are cornerstones in the management of HFrEF. Before reevaluating LV ejection fraction and considering implantable cardioverter-defibrillator (ICD) therapy, patients with newly diagnosed heart failure with reduced ejection fraction (HFrEF) outside of the context of an acute myocardial infarction are generally allowed to undergo a critical *waiting period* of approximately three months. This window of opportunity is provided to optimize guideline-directed medical therapy (GDMT) to induce LV reverse remodeling, which, if it occurs over a threshold, would eliminate the necessity for an ICD. Major professional groups support the idea of considering an ICD after this period [12].

This case highlights the importance of recognizing the cardiovascular risks associated with amphetamine use, and early detection and cessation of drug use to prevent further damage, particularly in younger patients who may be prescribed these medications for conditions such as ADHD. Given the potential for serious cardiac complications, clinicians should maintain a high index of suspicion for cardiomyopathy in patients presenting with heart failure symptoms who have a history of amphetamine use. Early diagnosis and intervention are critical to preventing irreversible cardiac damage and improving outcomes in these patients.

## Conclusions

This case of a 32-year-old male diagnosed with severe heart failure caused by amphetamine-dextroamphetamine-induced cardiomyopathy highlights the significant risk of cardiomyopathy and heart failure associated with prolonged amphetamine-dextroamphetamine use, particularly in young adults without cardiac risk factors. The development of nonischemic cardiomyopathy in this patient underscores the potential for stimulant medications, commonly prescribed for ADHD, to induce severe cardiac complications. Early recognition of the signs and symptoms of heart failure, as demonstrated in this case, is crucial for intervention and management. Although extensive literature exists on the subject, numerous uncertainties persist concerning the optimal management and treatment of patients with amphetamine-induced cardiomyopathy, emphasizing the necessity for additional research and the establishment of standardized clinical protocols. Drug abusers frequently receive inadequate attention to their physical health as a result of the historical compartmentalized approach to treating those with substance use disorders and addiction.
